# Generalized box-plot for root growth ensembles

**DOI:** 10.1186/s12859-016-1445-3

**Published:** 2017-02-15

**Authors:** Viktor Vad, Douglas Cedrim, Wolfgang Busch, Peter Filzmoser, Ivan Viola

**Affiliations:** 1 TU WIEN, Karlsplatz 13, Vienna, 1040 Austria; 2 ICMC – University of São Paulo, São Carlos, 15260 Brazil; 3 Gregor Mendel Institute of Molecular Plant Biology GmbH, Dr. Bohr-Gasse 3, Vienna, 1030 Austria

**Keywords:** Uncertainty visualization, Bioinformatics visualization, Curve ensembles

## Abstract

**Background:**

In the field of root biology there has been a remarkable progress in root *phenotyping*, which is the efficient acquisition and quantitative description of root morphology. What is currently missing are means to efficiently explore, exchange and present the massive amount of acquired, and often time dependent root phenotypes.

**Results:**

In this work, we present visual summaries of root ensembles by aggregating root images with identical genetic characteristics. We use the generalized box plot concept with a new formulation of data depth. In addition to spatial distributions, we created a visual representation to encode temporal distributions associated with the development of root individuals.

**Conclusions:**

The new formulation of data depth allows for much faster implementation close to interactive frame rates. This allows us to present the statistics from bootstrapping that characterize the root sample set quality. As a positive side effect of the new data-depth formulation we are able to define the geometric median for the curve ensemble, which was well received by the domain experts.

## Background

One of the largest challenges in biology is relating the genotype (i.e. the configuration of genes in the genome) to the phenotype (i.e. the properties of living systems, e.g. the physical appearance of an organism). In the past years, there have been major breakthroughs to efficiently determine the genotype using next generation sequencing technologies. Moreover, in several fields, such as plant biology, major progress has been also made to efficiently and accurately quantify the phenotype [1, 2].

In particular, the root is frequently the subject of systematic and large-scale phenotyping, as its growth properties are of major importance for plant productivity, but has not been the target for extensive breeding efforts. As a simple model, the root of the model plant Arabidopsis thaliana plays an important role in the root phenotyping community. This is not only because Arabidopsis is a small plant that can easily grow in large numbers in vitro to allow for efficient image acquisition of the root, but is also a great object for transgenic approaches [3, 4], it has large mutant collections [5], and many sequenced genotypic variations are available [6].

The aim of Arabidopsis root phenotyping experiments is to observe the growth of as many roots as possible with various genotypes. A large-scale phenotyping pipeline has been recently developed [2] allowing for the acquisition of thousands of root images per day in very high resolution and over subsequent days. Seeds are placed on the surface of agar plates that are filled with a nutrition gel. Each compound of that gel is very accurately controlled, so that each plant has the same conditions to develop. The placement of the seeds are guided by a predefined grid layout, so the genotypes of each particular plant can be traced back during analysis. Apart from image acquisition events, the plates are kept in a growing chamber, in controlled conditions.

The resulting images are subjected to image-processing based analysis methods for measuring traits (e.g. length, tortuosity, growing angle at a specific timestamp). While the computational analysis of these and other root phenotypic data is developed well [7–10], there is no tool allowing to visually explore or compare the complex phenotypes of multiple individuals or their statistical properties. Moreover, with hundreds of thousands of images and different genotypes distributed to many images, it is a challenge to conduct visual checks or to produce figures for presentations of specific genotypes. Finally, after publication of such data, the current method to share visual (non-abstracted) root data is to mail complete hard disks of images upon request making data sharing bothersome. These problems are amplified when working with time series of such complex data.

In this work we propose a system that supports root growth phenotyping of young seedlings of *Arabidopsis thaliana*, being designed for analyzing its earlier stages when the root individuals have not developed lateral branches. The domain experts usually use that early-staged analysis when they are interested in comparing geometric properties of the root individuals (length, growing angle, etc.). A simplified explanation of root phenotyping is the following. The researchers group individuals according to their genotype. In our test dataset a typical group has 10-15 individuals, but sometimes less, and in extreme cases many more is possible. For each individual in group traits are measured. Then, statistics (e.g mean) are used resulting in one trait representing a genotype. Finally Genetic Wide Associaton Mapping helps to reveal statistical relevance between the measured trait and the genetic sequences. This statistical approach already led to significant results, however it does not convey the characteristics of processed data.

In this paper we focus on a visualization system, which helps characterize the phenotypicical expressions, grouped by a common property (same genotype or same growing environment).

In root phenotyping it is common to measure 15-20 individuals per group. However, according to the knowledge of the authors, the question was never approached if 20 individuals per group is enough for a valid statistics in root phenotyping. Our system allows the domain researchers to visually explore many individuals which are grouped by their genotypes. This allows for the generation of highly informative figures for presentation and publications that go beyond root trait averages and make it possible to intuitively asses heterogeneity between different individuals.

### Related works

The shape variability between roots of one genotype can be analysed with respect to different properties. One possible way is to consider each root as a curve in a two-dimensional space. Since each root (i.e. curve) represents a possible different growth pattern, which defines the source of uncertainty in this context, one can make use of descriptive statistics to analyse the uncertainty associated to the process.

Graphical representations of one dimensional descriptive statistics (e.g. boxplots) have been widely used for statistical analysis in many different fields. Its main purpose is to visually convey statistical quantities estimated from the data with few assumptions on the underlying distribution. Several generalizations for multivariate statistics have been proposed. Bagplot [11], one of the earliest point-wise generalizations, uses convex hull peeling to convey the notion of centrality, spread, correlation, skewness and tails of the estimated distribution. Distance based depth functions (e.g. Mahalanobis depth) are also a classic estimation strategy with the additional property of being simple to implement, while achieving good centrality estimates [12]. It has been shown that carefully aggregating many different plots into a single one can be an effective way to summarize such statistics in a meaningful way, while avoiding cluttering [13].

From the data perspective, the linear root growth is highly related to trajectory data, like simulated ensemble of hurricane trajectories [14, 15]. The shape variability of the trajectories ensemble exposes the need for a better understanding of the underlying uncertainty of the simulation process.

In the scientific visualization literature there have been many successful attempts to provide algorithms for understanding uncertainty through visualization. They are able to deal with different kinds of complexity such as: high dimensionality of the data, time series and ensemble simulations. In this sense, the visualization contribution of our work can be categorized as uncertainty visualization of ensemble of functions [16, 17].

Cox et al. [14] propose a visual representation of path ensembles in the context of hurricane forecasts. However, while it can lead to a cluttered and sometimes overcrowded visualization, its aggregation strategy can nicely convey the notion of shape variability among one ensemble of curves. We use a similar strategy as one part of our system by aligning the roots to a common starting point, which allows for the domain experts to explore the variability of shapes within each genotype.

In a similar context of weather forecast models, Sanyal et al. [18] propose a visual metaphor combining 1D colormaps with variable sized circular glyphs in order to encode variability among ensemble members, which does not allow for easily comparing different ensembles and also does not take into account time dependency.

The recent usage of Radial Basis Functions (RBFs) with adaptive bandwidth selection, in the context of hurricane prediction, improves the smoothness of such visualizations by interpolating a simplicial depth sampled from its path ensemble [15]. Apart from leaving some parameters to be set by the user, their strategy is costly since, for each time step, they need to solve linear systems with the order equal to the number of samples, then to estimate minimum enclosing ellipses, and to perform a nonlinear filtering for smoothing its time step trajectories.

In descriptive statistics the *data depth* conveys the notion of centrality of the samples within the data distribution. In order to perform such estimates, one needs, among other things, to define the notion of center-outwards ordering, called data depth, where the "deepest" sample is the closest to the center. In the 1D case it is straightforward to do so by using a simple ordering of the samples, and using the distance to the median as the centrality measure. There are many different ways on generalizing this concept to higher dimensional samples (e.g. simplicial depth)[12], to curves or functions (e.g functional depth).

Sun et al. generalized data depth to 1D functions defined on a common interval. The authors defined bands with J samples, J at least two. A band consists of functions, chosen from the ensemble. A function lies in a band, if for the entire common domain interval its value can be bounded by other values coming from functions from the band. A data depth of a function is defined as the estimated probability of the function-in-question lying inside a band. It was also proved that computing band depth with iteratively increased J, than summing up the result gives a more robust estimator.

Mirzargar et al. took the aforementioned method, and generalized it to multidimensional curves [19]. In their approach a curve lies in a band, if for the entire domain interval its points are inside the convex hull of the points, which are evaluated from the curves of the band.

In the aforementioned methods the most representative sample (function/curve) is chosen to be the one, which has the highest depth value, consequently it is the most “central” one. As we show it with examples, this approach works well if there are enough samples for the analysis.

## Methods

### Motivations

Biology researchers use numerical traits to describe root ensembles. These traits are crucial to understand the underlying phenomenon. However, these biological traits describe only one aspect of a root ensemble, therefore it is difficult to give answers for questions which relate to the behavior of the root ensemble as a whole. Before thorough analysis it could be very useful to explore:

- What are the growing trends in the ensemble?
- What is the variability of the ensemble?
- What could be a typical root in ensemble?
- What is the statistical quality of the ensemble?
- Is the acquisition feasible at all?

We were looking for a Visual Descriptive Statistics, which addresses some of these requirements.

Our system is tailored for early-staged phenotyping (the root individuals did not start developing branches), and the traits used in these experiments aim to describe the shape of the root individuals. Moreover, our domain experts claim that if a root branch (including the main root) already developed a shape, that part will not change in future in these in-vitro experiments. In other words, to model the shape of the main root, it is enough to represent the time dependent position of the root-tip. Putting the aforementioned detail under consideration, we decided that an early-staged growing process of a root individual can be modelled as a time-dependent parametric planar curve.

**Fig. 1:**
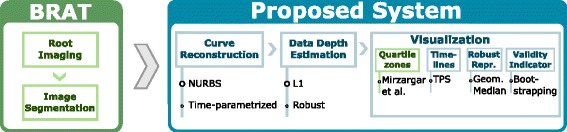
On overview of our system pipeline. *Green* indicates imported technologies or methods

An overview of our system pipeline can be seen at Fig. 1. Our system is a visual analysis frontend of the Busch-lab Root Analysis Toolchain system (BRAT), which was developed by Busch et al. [2]. For our methods we turned the representation of root individuals from pixel-based to a curve-based one. Our visualization components (Quartile zones, Timelines, Representative curve, Validity indicator) show various statistical aspects of a curve ensemble. For the spatial-variability visualization a centrality estimation is needed. This is achieved by our robust *L*_1_ data depth estimation component.

The BRAT toolchain outputs a subset of segmented pixels, where every pixel belongs to a root individual. For visual analysis we turn the pixel-wise representation into B-Spline curve-based representation, where each curve represents one root individual. The knot vector (which determines the B-Spline basis) and control points are stored in an SQL database. During visualization stage, according to the users needs our client application reads these parameters for the requested root individuals, and forms a curve ensemble for further analysis. Every proposed method in this paper takes these B-Spline curve ensemble as its input.

**Fig. 2:**
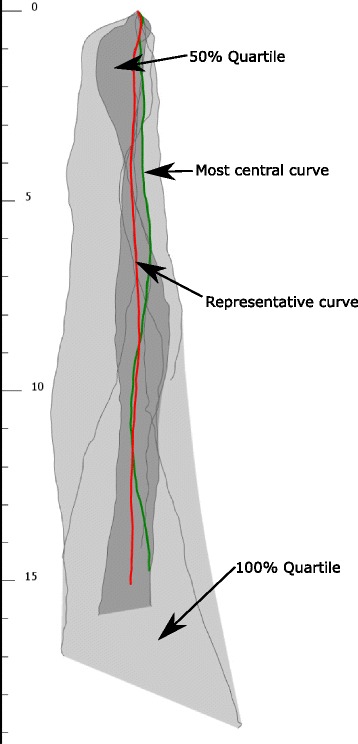
Concept of Curve Box Plot. *Dark gray* zone represents the 50% quartile, *light gray* zone represents the 100% quartile. *Green curve* is the most central *curve*, *red curve* is the proposed representative *curve*

For our visual statistics of curve ensemble we have chosen the Curve Boxplots (Fig. 2), which is a recent work of Mirzargar et al. [19]. On one hand our system heavily relies on it, and uses similar visual design for spatial quartile zones. On the other hand due to the special manner of our application we had to introduce changes and extensions:

- A more robust curve data depth estimator
- A robust ensemble representation
- Time-lines with confidence zones
- Ensemble validity indicator

The key element of a visual statistical method is the choice of data depth. In case of functional data (curves) it is most commonly based on band data depth, as it was the case also in Mirzargar et al. [19]. However, in our application it occurs many times that the size of a root ensemble is rather low. In these cases the band data depth is not sensitive enough for the relative distances of the curves. Furthermore, occasionally in these cases the usual way of picking a representative curve (the most central one) does not seem to work properly. So we propose a new representative curve estimation, which could fit better to the small sized ensemble intuitively.

In our application we followed the same method as in the aforementioned works for determining inter-quartile, and inlier zones. With data depths we have an ordering. The median of the depth values is computed, and samples, whose depth values fall into the range of the highest depth value and the median depth value are considered to lie in the inter-quartile zone. A threshold for inlier/outliers is also computed, as the length of the inter-quartile depth range multiplied by a chosen scalar. For this scalar parameter we chose 3 in our application (just as in [19], suggested by Tukey).

Curve Box-Plots only tell us whether a spatial position has a probability to be occupied by a root, but it does not say anything about when it could be reached. However, growth time is a crucial information for our domain experts, so we designed a representation to convey that information for the user. In root phenotyping time and growing speed is an essential property, which is analyzed thoroughly. Therefore it was vital for us to develop a visual representation, which could informatively express the overall growing speed, and its deviation in the ensemble.

### Reconstruction of roots as curves

Our system was built on the top of the Busch-lab Root Analysis Toolchain system (BRAT), which was developed by Busch et al. [2]. The BRAT system allows for acquiring the images and creating binary segmentation. BRAT root segmentation framework first detects plants by finding their shoots (green area). Then it is assumed the edges of roots can be detected in the proximity of shoots by finding highly saturated edges (using Sobel filter). Then the main root pixels are estimated with skeletonization.

Representing roots with their raw segmented pixels has some drawbacks. Due to the high resolution of scans these pixels can be numerous, even if the represented root is relatively smooth. In addition, the segmentation results can be noisy. Therefore we chose to use a parametric representation, namely Non-uniform B-Spline curves [20]. B-Splines are widely used in many fields, including CAGD and statistics. They are flexible piece-wise polynomial representations, which can describe complex free-form shapes with relatively few parameters. In addition, with the right number of control points, a B-Spline representation can smooth out the noise while keeping the complexity of shape in question. Moreover, since it is a parametric representation, it allows a visually appealing rendering.

Each root representation estimation starts with the list of segmented pixels (samples), which belong to the same underlying individual root. One of the pixels is selected as the starting point (i.e. it is the closest one to the detected seed of the plant). First parameter values have to be assigned to every discrete pixel. For that the segmented points have to be ordered, which is not necessarily straightforward due to many segmentation errors. We used a pixel-based Dijkstra-algorithm, starting from the chosen start point, and the assigned parameter values are the resulting Dijkstra-distances, normalized to [0,1].

Choosing the correct number of control points is important: using too few will over-smooth, using too many will over-fit the noisy data. We used a conservative measure proposed by Yuan et al., which is based on Fourier Analysis of the B-Splines [21]. Their argument was, that the lower bound of the number of needed control points is $\#CP\geq \frac {\#samples}{4\pi (b-a)}$, where the B-Spline’s domain is [*a,b*]. We fit our curves to domain of [0,1], and we empirically selected $\#CP=\frac {1.2\times \#samples}{4\pi }$. With this choice the number of control points is above the lower bound, but close enough to avoid over-fitting. The knot selection comes from deBoor’s classic knot selection algorithm [20]. It ensures, that the knots are chosen in a way, that the number of samples falling into curve segments are relatively the same.

**Fig. 3:**
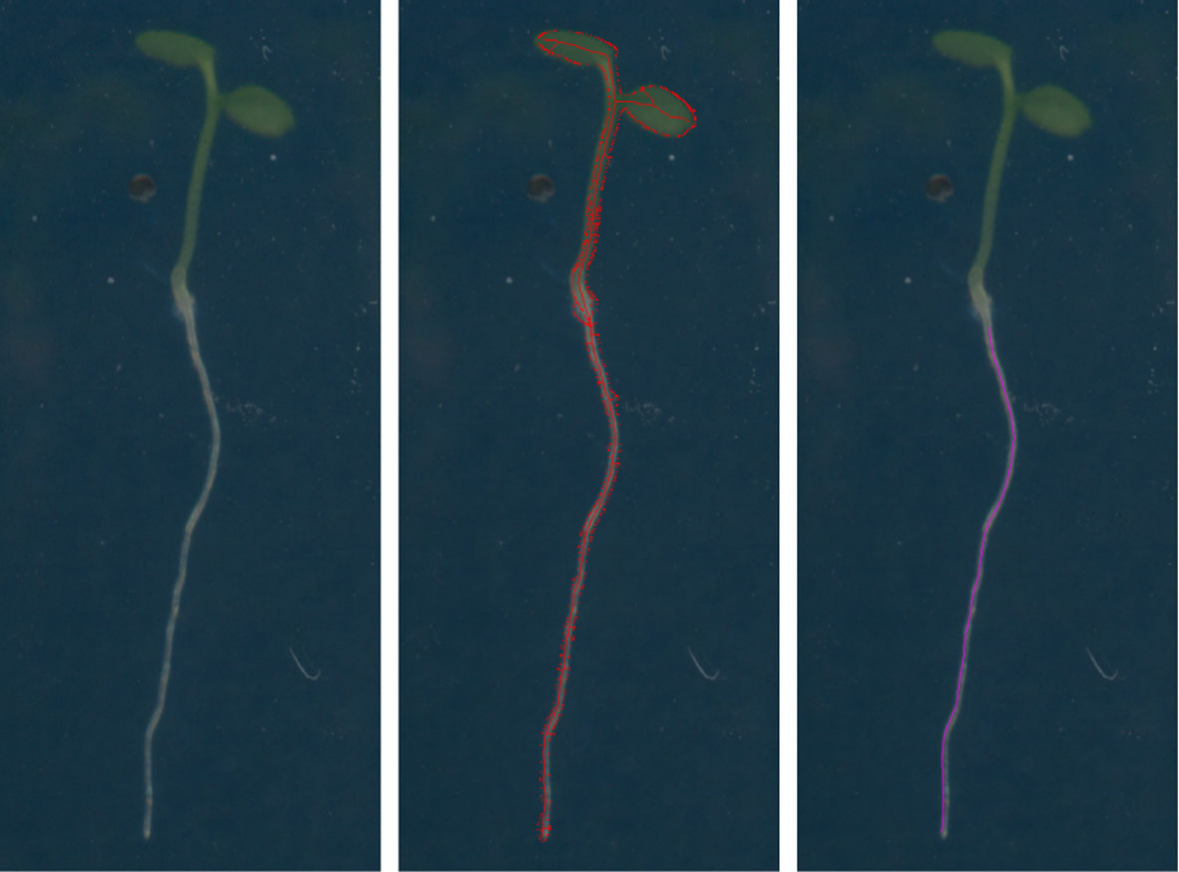
On the left there is an example of acquired image of a root. The results of pixel level segmentation are shown in the middle. While on the right, reconstructed curve is shown

Then a Least-Squares optimization is performed, determining the control point coefficients. We used 4th order (3rd degree) curves. As a result we represented a root object as a continuous parametric curve in the image domain. However, our intention is to represent the time-dependent growth of root individuals with curves, not only their shape. Therefore we re-parametrized our B-spline curves from [0,1] to [0,*d*], where *d* stands for the day when the image containing the segmented root individual was acquired. Despite it is a rough time-parameter approximation, and only accurate in daily unit, our domain experts agreed that it is enough for their purposes. However, technically more accurate estimation is possible. A raw acquired image of a plant, segmented pixels and reconstructed root-curve can be seen on Fig. 3.

### Visual representations of root ensembles

Our visual representations take root ensembles as inputs, where a root is modelled as a B-Spline curve. The visualizations represent various statistics of curve ensemble, therefor they can be seen as parallel functionalities in our pipeline.

### *L*_1_ data depth

For Curve Box-Plot visualization, which represents the spatial variation of the ensemble data, a proper data depth estimation step is responsible for measuring the centrality of each curve. However, as it was mentioned before, the high variability in size of our ensembles makes it necessary to apply a robust data depth estimation.

Vardi et al. [22] introduced a robust data depth for multidimensional Euclidean point-set, that they called *L*_1_ data depth. Their formulation stems from the solution of a completely different problem, namely the Fermat-Weber location problem.

The geometric median (multivariate *L*_1_-median, spatial median) is the theoretical solution of the Fermat-Weber location problem.

In general case the geometric median is estimated by a simple iterative method, the Weiszfeld algorithm. Vardi [22] pointed out a “leak” in the original classic method, and proposed the modified Weiszfeld algorithm.

More importantly, the authors introduced a depth function, which they called *L*_1_-Depth. It can be seen that the a normalized weighted sum of the distances to the input samples is assigned to each position of the input space.

It is important to note, the point that maximizes the *L*_1_ data depth for a given point-set (therefor it is the most “central” point), is the fixed point of (modified) Weiszfeld algorithm, the geometric median. A great advantage of *L*_1_ data depth is its efficiency to calculate. However, a greater advantage for our application is that it is very stable, even for small number of samples. For further details and definitions please refer to [22], or Appendix A for a summarized version.

### Applying *L*_1_ data depth to B-Spline curve ensemble

One of our main statements in this paper is that *L*_1_ data depth, which is so far defined on Euclidean space can be exported to our B-spline representations.

So far, all curves in the ensemble are defined by different basis functions. In order to make them comparable, we have to convert them into a common basis. First, we check what is the maximal number of control points our curves in the ensemble have, that will be the control point number of the common basis. Then, we concatenate all knots of every B-Spline system in ensemble into a temporary buffer. After sorting and removing repeated knots, we define a new knot sequence with the already mentioned deBoor’s method. Each original curve is evaluated in several discreet parameter values, falling into the interval of the common B-Spline basis. Then with Least-Squares fitting we get the new curve in the common basis.

In this paragraph we claim that a B-Spline function is well defined by its coefficients. Also, it is easy to see that these B-Spline functions Hilbert-space distances can be approximated by their coefficients Euclidean distance. For simplicity lets consider 1D B-spline functions at this moment, which are spanned by the same bases *B*_1_…*B*_*n*_ (Since B-Spline curves are defined by B-Spline functions as coordinate functions, it is straightforward to see that similar argument is true for them). The functions are also members of the Hilbert-space with *L* ^2^ scalar product. Lets define an operator



which maps the coefficient vector to its B Spline function. It is known, that *T* is a linear bounded operator. Also, we can define its adjoint operator



which is also known to be a linear bounded operator. The proof for generic cases (infinite dimensional Hilbert-spaces) can be found in Chapter 2 in [23], as Lemma 2.1. Assuming that *f* is a result of Eq. 1 for some coefficients *c*_1_…*c*_*n*_, these original coefficients can be traced back by solving the system *G*[*c*_1_…*c*_*n*_]^*T*^=*T* ^⋆^(*f*), where *G* is the Gramm-matrix of *B*_*i*_-s. The boundedness of the coefficients comes from the fact that G is a positive definite d-band matrix, where d is the order of the B-Spline basis.

Our data depth estimation for Non-uniform B-Spline curve ensemble is simple. We re-define our B-Spline curves into the same basis. Then for each curve we concatenate its control points coefficients, so we represent each curve as a 2×*n* dimensional Euclidean vector. We compute the *L*_1_ data depth for these vectors, and we can assign these depths as the data depths of the curves.

### Geometric median curve

The state-of-the-art methods choose the most central curve as a representative. In this way the representative curve is part of the ensemble.

In this section we propose another possibility. After we computed the *L*_1_ data depths each curve is represented as a 2×*n* dimensional vector. We compute the geometric median of these representative vectors. However this geometric median vector also represents a B-Spline curve. As a consequence of the linear boundedness property of Eqs. 1 and 2, the geometric median based curve approximately inherits the “most central” property from the Euclidean vector representation.

### Time information estimation

Since our curves represent a time-dependent root growth, visualizing time is also an important issue. For time visualization we chose to use a design, where we show a continuous “front-lines”, or time-lines for different time values (days, in our application), also with their confidence zones. These confidence zones of time-lines deliver a visual clue about the variance of the statistical estimation of the particular time-line. It can deliver information about how diverse the growing speed of the rootsin the ensemble is.

A naive approach would be to estimate a smoothing spline for every time-line separately from the evaluated points of root-curves. However, in that way we would face technical difficulties, like parameter estimation, but more importantly we would not be able to ensure that the splines would not “tangle” to each other.

**Fig. 4:**
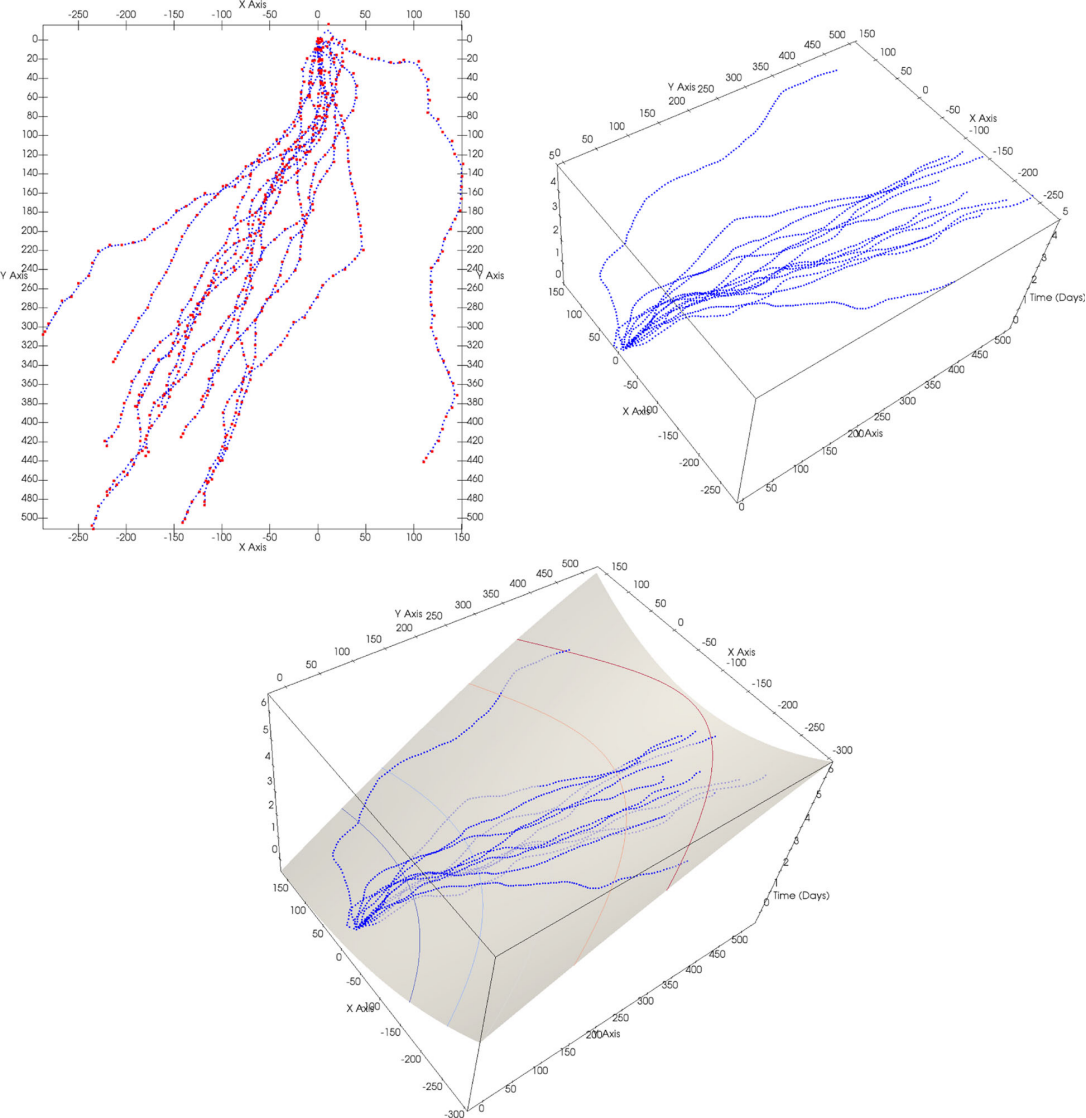
*Top left*: Input 2D sample (*blue*), with control points as centers of TPS (*red*). *Top right*: Input samples the time value as 3^*rd*^ coordinate. *Bottom*: Fitted TPS surface with its isocurves, representing front-lines

We followed a different approach. Lets assume that *t*_1_,…,*t*_*M*_ are discreet values in the (time) domain of ensemble. The curves in the ensemble are evaluated in all *t*_*i*_,*i*=1…*M*, resulting in $\mathbf {p_{i}}\in \mathbb {R}^{2},i=1\ldots M$. We are looking for an interpolating smooth surface $F(\mathbf {x}):\mathbb {R}^{2} \to \mathbb {R}$, *F*(*p*_*i*_)=*t*_*i*_,*i*=1…*M*, as shown in Fig. 4. For this task literature offers many possibilities for statistical surface interpolation (B-Spline surfaces, Local Linear Regression, Moving Least-Squares, etc.).

Thin Plate Spline (TPS) surfaces are widely used in a variety of statistical applications, like machine learning, or non-linear support vector machines [24]. It is an interpolating surface with the property, that among all other smooth surfaces it minimizes the functional $\iint _{\mathbb {R}^{2}}\left [\left (\frac {\partial ^{2} F(\mathbf {x})}{\partial x^{2}} \right)^{2} +2\left (\frac {\partial ^{2} F(\mathbf {x})}{\partial x\partial y} \right)^{2} +\left (\frac {\partial ^{2} F(\mathbf {x})}{\partial y^{2}} \right)^{2}\right ]d\mathbf {x}$. This minimal property ensures us that the time-lines are not crossing each other.

A Thin Plate Spline is defined in the form

3 $$  F(\mathbf{x})=\beta_{0}+\beta^{T}\mathbf{x}+\sum_{j=1}^{N}{\alpha_{j} \phi_{j}(\mathbf{x})},  $$

where *β* are coefficients for linear part, *α*_*j*_,*j*=1…*N* are coefficients for the non-linear part, which are determined by kernel functions *ϕ*_*j*_=‖**x**−**c**_*j*_‖^2^ log(‖**x**−**c**_*j*_‖). Here, the $\mathbf {c_{j}}\in \mathbb {R}^{2}$ are pre-established centers. The linear and non-linear coefficients are found with Least-Squares fitting.

The reader can notice that estimation of TPS surface does not need any other parameters (e.g kernel width) beyond centers.

In literature there are two approaches how to determine centers. The “classic” way is that samples *P*_*i*_ serve as centers. However, it produces a huge linear system to solve. Also, *ϕ*_*i*_(**0**)=0, therefore the matrix of the linear system would have a zero diagonal, which would lead to a extremely badly conditioned system. A second approach is to define centers as points on grid, considering only those which are inside the convex hull of input samples. However, that needs predetermining the grid size as an initial parameter, and also involves inclusion test for each grid points.

We decided to take advantage of our Non-uniform B-Spline representations. We used the control points of the curves as centers. This approach is resolution-less (unlike the grid version), however the control points are still “close” to the input samples (evaluated curve points). In addition, the formulation of the resulting TPS will not depend on *M*, the discretization parameter of the curves. We got very similar results to the one with using sample points as centers.

The time-lines are rendered as isocurves of the TPS surface in the planar domain (see Fig. 4).

We also wanted to convey a confidence zone for time-line. For that, a variance had to be computed for each timeline isocurve. Every curve in ensemble was evaluated at the time value in question. Then, the spline surface was also evaluated, getting the estimated time value for each position of the curve. The variance is computed from the differences of the expected time value and the evaluated time values.

**Fig. 5:**
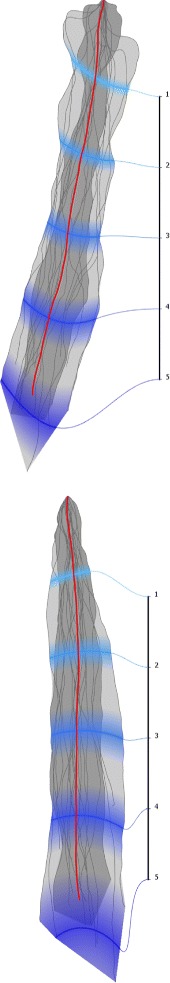
Examples for Time-line visualization for different genotyped ensembles, with error zones. The technique immediately conveys diversities in growing speed among the ensemble. *Top*: Ensemble with 21 curves. *Bottom*: Ensemble with 21 curves

Time-lines are rendered as isocurves of the fitted TPS spline. For each timestamp (days in our examples) we strike the isocurve of to the timestamp value. The corresponding confidence zone is rendered by alpha blending, linearly decaying toward the border of the zone. The time-lines are connecting to a timestamp indicator ruler continuously. In addition, each time-line is rendered with a unique color coming from a colormap. We chose a colormap based on color blue, where brighter blue colors indicate earlier timestamps. As examples in Fig. 5 show, this technique immediately conveys diversities in growing speed among the ensemble.

### Validity indicator of median estimation

In this section we propose a visual representation, which conveys information about how feasible our representative curve estimation is for the given ensemble. Basically we compute a spatial confidence zone for the representative curve estimation, with respect to a “little” change in the consistency of the given ensemble. Since representative curve estimation is a robust deterministic method, we had to borrow a statistical method, called bootstrapping.

Lets define *E*={**x**_1_,…**x**_*m*_} as the curve ensemble represented by Euclidean vectors (see previous Section). Then, we can define *E*_*i*_={**x**_*r*1_,…**x**_*rm*_}, where *i* is a number between 1 and *B*, where *B* is the bootstrap samples, a predefined parameter. Also, *rj* is a random integer between 1 and *m*. These randomly picked indexes do not necessary have to be distinct, and also it is allowed that they do not cover all numbers between 1 and *m*. Then, since *E*_*i*_ is also an ensemble, we can compute its geometric median representation curve. Lets denote that as $\hat {\mathbf {x}}_{i}$. If we repeat this computation *B* times, we can collect all $\hat {\mathbf {x}}_{i}$ into a new ensemble $\hat {E}$, consisting *B* vectors (curve representations). If we compute the *L*_1_ data depth for $\hat {E}$, the corresponding curves inter-quartile zone represents the spatial zone, which we call validity indicator of median estimation.

Although we present this visual representation for a particular application, since bootstrapping is a general method, similar approach could be used to visualize the feasibility of other median/representation estimator.

## Results and discussion

### Implementation

We implemented our methods in C++, with the utilization of Eigen and Boost libraries. Visualization was implemented in OpenGL.

For visualization of the ensemble, roots are represented by non-uniform B-Splines, whose control points are in image space, scaled that one unit means one pixel. We found it informative if we showed a ruler with units in mm. The metric length could be computed from the dpi resolution of the scanned image, whom the root individual was acquired from.

For comparison we also implemented band data depth for curves, as it was presented in work of Mirzargar et al. [19]. Since our curves (represented roots) are extremely irregular, for band depth we were forced to use the technique, which the authors called modified band depth. In this case the a curve’s inclusion in a band is not a binary function, but relative length of parameters where the curve lies inside the band.

**Fig. 6:**
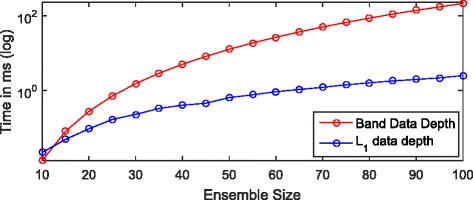
Computational speed for data depth estimations

Initially we compared the computational speed of the two algorithms. In order to create a fair algorithmic comparison, we did not use multi-core optimization for either methods. We evaluated both methods for systematically increasing ensembles, where the individuals were randomly selected. For band data depth we used 128 discrete samples for curves. Figure 6 shows, that for smaller sizes, the computational speed is comparable. However as size increases, the needed computational requirements became higher by orders. However it is also fair to mention, that band data depth is easily parallelizable, while for *L*_1_ data depth it is not that straightforward to parallelize.

### Geometric median curve

**Fig. 7:**
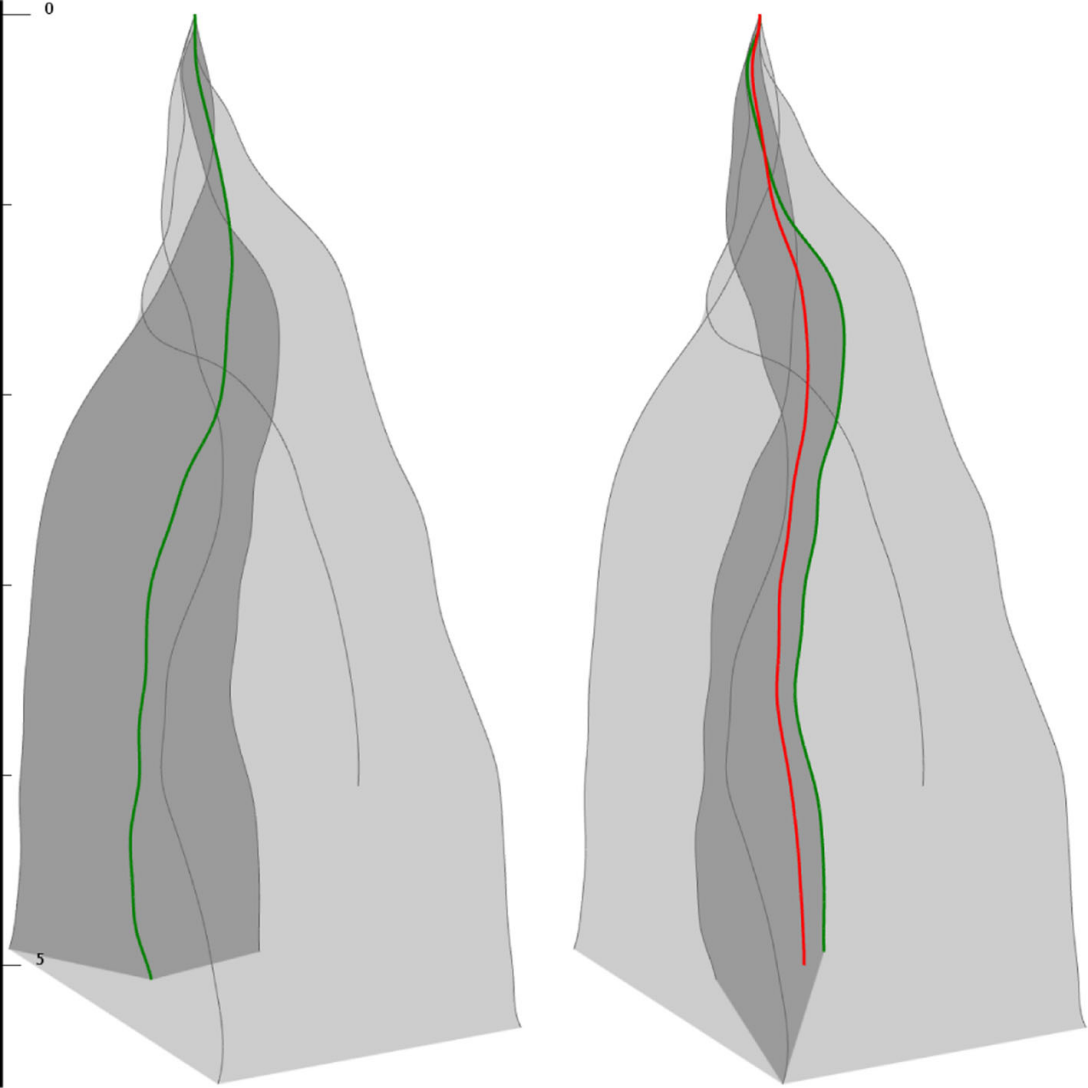
Comparing boxplots for the same curve ensemble (6 *curves*), but estimated by different methods. *Green curves* represent the deepest ones, *red curves* show the geometric median-based ensemble representer. *Left*: Mirzargar’s method. *Right*: Proposed method

**Fig. 8:**
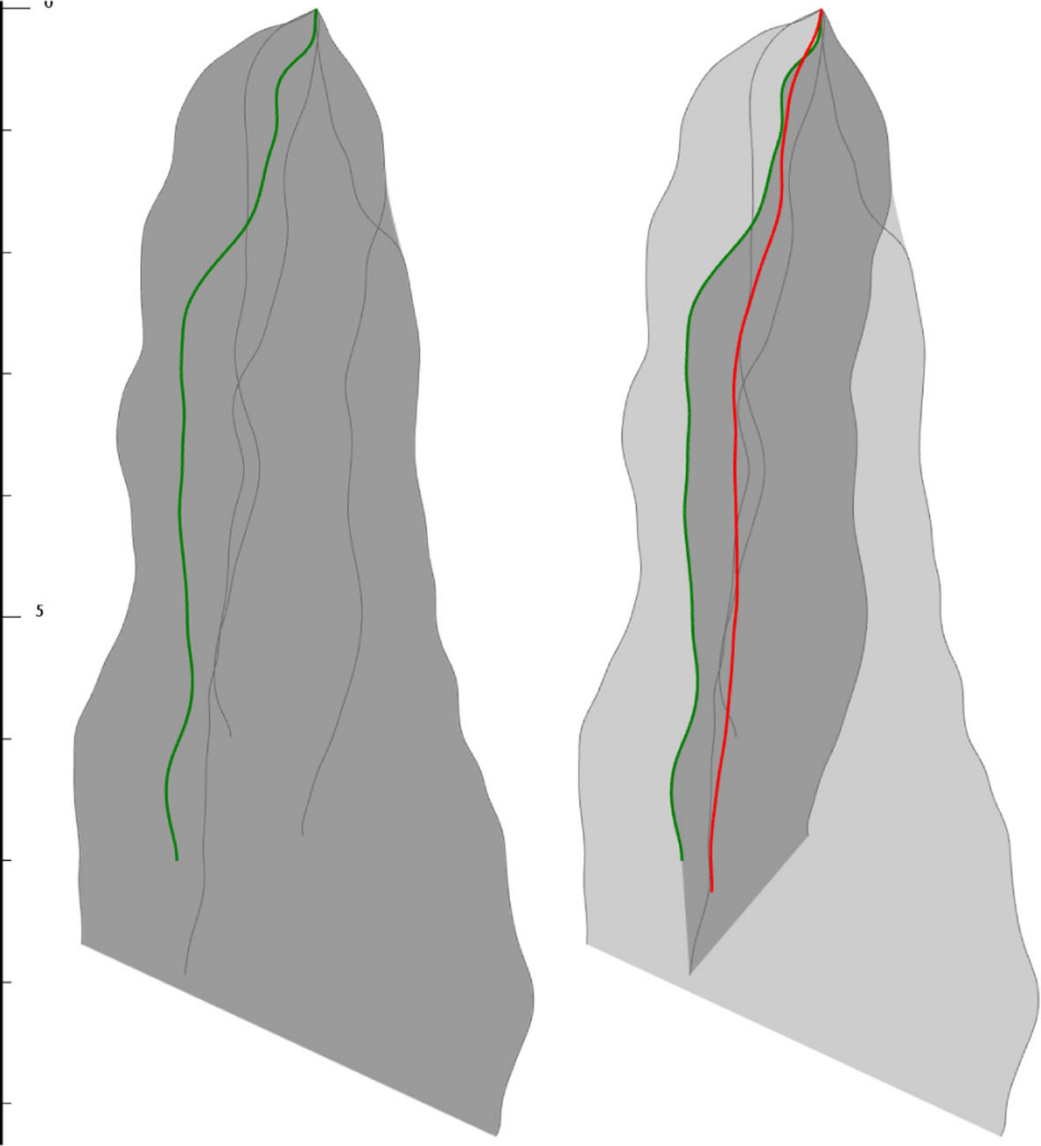
Comparing boxplots for the same curve ensemble (6 *curves*), but estimated by different methods. *Green curves* represent the deepest ones, *red curves* show the geometric median-based ensemble representer. *Left*: Mirzargar’s method. *Right*: Proposed method

A big difference to the state-of-the-art is that the proposed representative curve is a virtual curve, it is not part of the original data ensemble (Examples can be seen in Figs. 7 and 8). Despite the proposed representative curve seems centralized, we had to verify its representation capability with domain experts. In addition we wanted to find out if our method compares better or worse to the existing state-of-the-art method. For that purpose we conducted a survey among domain experts with a comparative questionnaire.

In our survey we created a PDF form, which was spread by emails to domain experts internationally. The form started with an introduction, then it contained seven cases (placed on separate pages). Each case the same root ensemble were shown with two different versions of curve box plots next to each other. One version was estimated with Mirzargar’s box plots, using band data depth, and one visible curve indicated the deepest one as representative. The other version was also with curve box plots, using the proposed *L*_1_ data depths, and the representative estimated with our geometric median based method. The order of methods were randomly chosen. The experts had to rate from 1 to 5, which version they found more representative. A rating of 1 meant the expert found the left plot more representative, while 5 indicated the same with right one, and 3 meant no significant difference.

We scored the answers of the questionnaire. An answer got score in 5 scales according to preference of the expert, ranging from “absolutely the Mirzargar’s one” (-2) over “no difference (0) to "absolutely our one” (2). As it can be seen on Table 1, the effect is rather small. On the other hand an important observation is, that if the ensemble contained less than 10 samples, the experts preferred the geometric median curve over the band data depth median.

**Table 1:** Result scoring of our user study

*ensemble size*	7	7	14	8	16	9	9
Domain expert1	−1	−1	−1	−1	−1	−1	2
Domain expert2	2	2	0	2	−2	−2	2
Domain expert3	0	0	0	0	0	0	0
Domain expert4	−1	−1	−1	−1	−1	−1	0
Domain expert5	1	2	−1	−1	−1	1	1
Domain expert6	2	2	−1	1	−1	1	2
Domain expert7	−1	2	−1	1	1	1	1
Domain expert8	1	1	0	0	1	1	1
Domain expert9	1	1	1	1	1	−1	−1
Domain expert10	2	−2	−1	2	−1	1	−2
**mean**	0.6	0.6	−0.5	0.4	−0.4	0	0.6

The methodological preference for an ensemble was computed by averaging the domain experts scores for the ensemble. If the ensemble contained less than 10 samples, the experts preferred the geometric median curve

Despite that several domain experts found the new representation interesting (“In my opinion, the geometric median-based curve can represent the group’s overall position very well”), a smoothing effect over the representer curve has been noticed. In root phenotyping this is not desired, since some traits (tortuosity, linearity) are not represented well. This effect can be related with the proposed feature space and it requires a further investigation of its cause.

### Validity indicator

The validity indicator is defined as the inter-quartile of $\hat {E}$, as it was previously detailed. To avoid confusion with the spatial box plot, we decided to render the indicator as an outline curve.

**Fig. 9:**
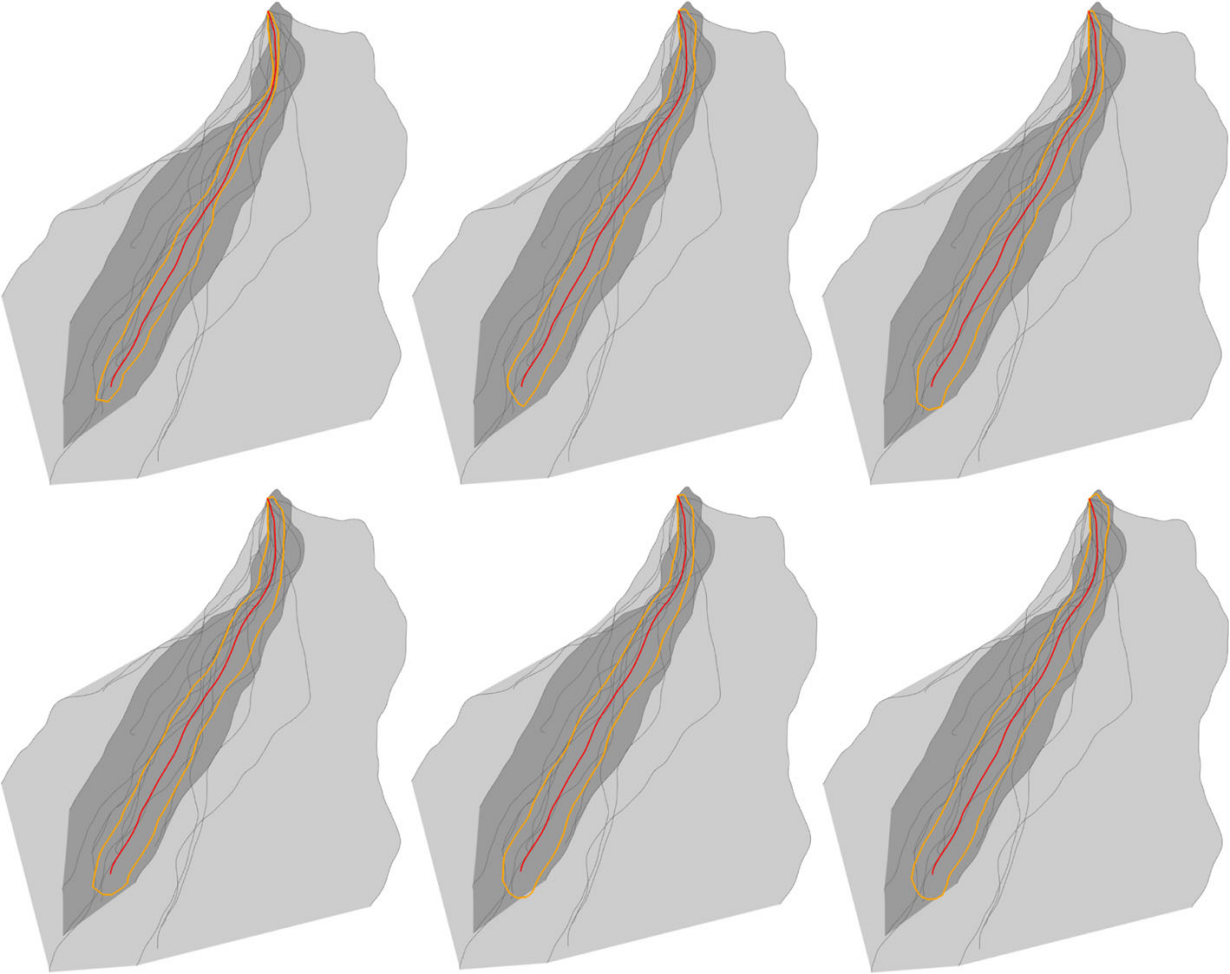
Showing geometric median curve validity indicator computed by different number of bootstrap iterations. Using more than 300 iterations does not change the indicator shape significantly. *Top left*: 25 iterations. *Top middle*: 50 iterations. *Top right*: 100 iterations. *Bottom left* 200 iterations. *Bottom middle*: 300 iterations. *Bottom right*: 400 iterations

A very important question may arise, namely what parameter should be chosen as the number of bootstrap iterations. We answered this question empirically. For some ensembles with relatively high diversity, we computed the validity indicator with increasing number of bootstrap iterations. Since bootstrapping is a stochastic method, too few iterations will cause changes in the shapes of indicators. Our decision was that for an ensemble with 15-20 individuals using more than 300 iterations does not change the indicator shape significantly. An example can be seen in Fig. 9. In that plot some changes in the shape of the indicator can be seen when few iterations were applied.

**Fig. 10:**
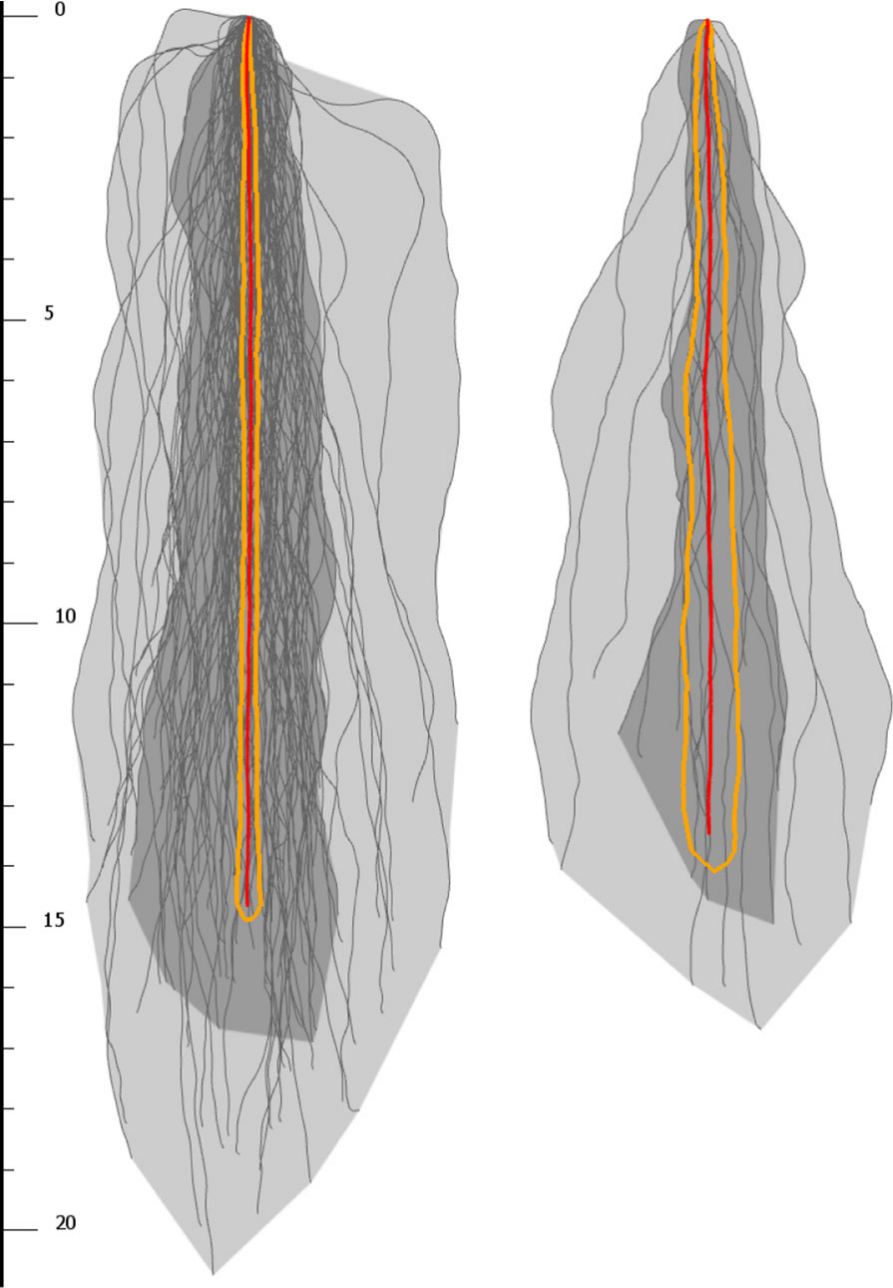
Plots with different ensemble size, with the same genotype. The area of validity indicator increases, as the size of ensemble decreases. *Left*: Ensemble with more than 100 curves. *Right*: Random subsample of *left*, consisting 20 samples

A straightforward hypothesis is that the area of validity indicator increases, as the size of the ensemble decreases. This hypothesis was confirmed by our experiment. We have a genotype, to whom around 100 individuals were segmented. For the full ensemble, and subsequent subsamples of them we estimated the validity indicator. Results can be seen in Fig. 10.

**Fig. 11:**
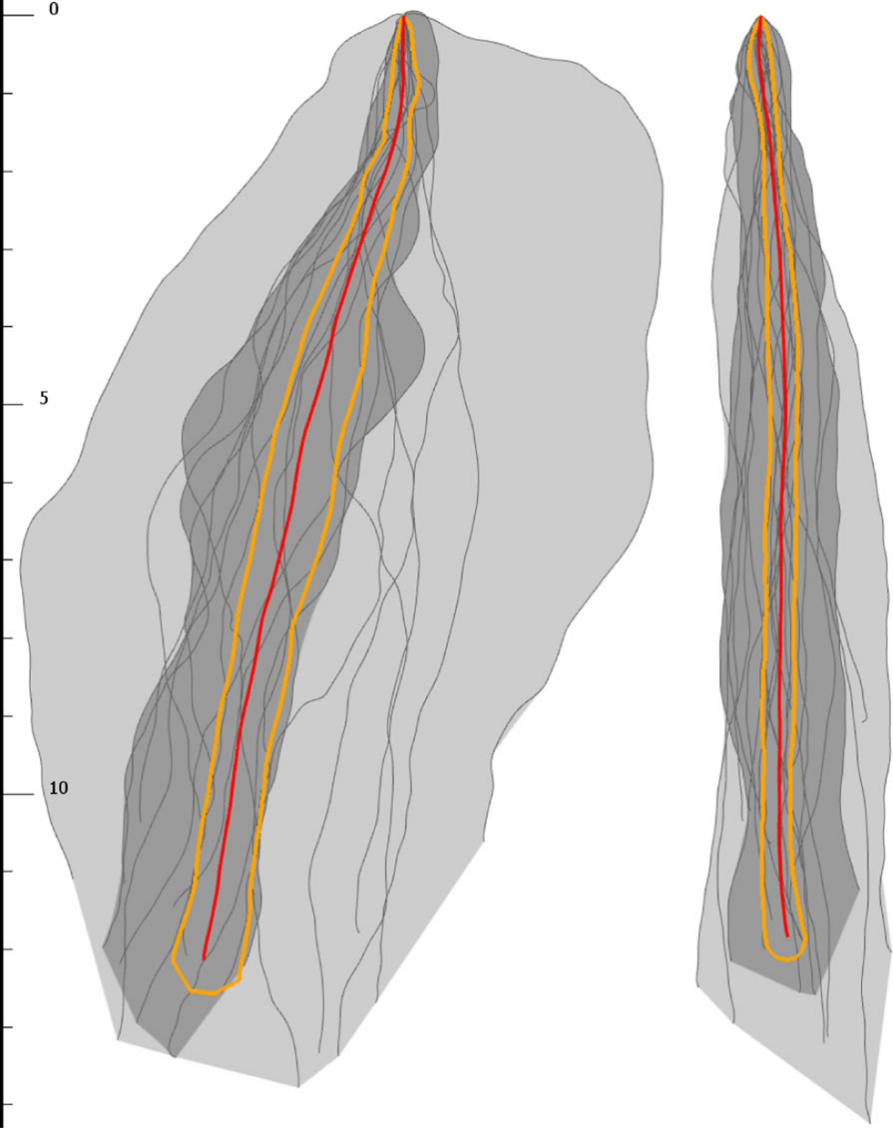
Plots with similar ensemble size, but with different genotype. The shape of the validity indicator can also be different for two ensembles with similar sizes. *Left*: Ensemble with 20 curves. *Right*: Ensemble with 21 *curves*

However, an interesting result is that the shape of the validity indicator can also be different for two ensembles, even if the ensembles contain around the same number of root individuals (see Fig. 11). This difference can only stem from the difference of the ensembles.

### Feedback from domain experts

During development, we consulted with domain experts in root phenotyping. As stated by them, this method allows efficiently to inspect and compare variation of root growth patterns. According to their knowledge, there is no other tool to efficiently do this. This enabled for the first time to efficiently visualize complex statistical root growth properties for the roots in multiple large datasets that already exist. It allows for a quick visualization that would be otherwise take hours to assemble using the current workflows.

The visualizations of a large set of accessions led the domain experts to the immediate insight that there is a genetically determined control of variance for root growth that seems to be time dependent. Some genotypes display control of root growth variation strictly throughout the five days of the time-course while other only do it during specific time-points; other genotypes don’t control for root growth variation very much. These observations indicate that the parameters for the visualization seem to be suitable for genetic mapping to identify genetic components that control this root growth variance.

## Conclusions

Presented workflow has taken as input images with segmented pixels of root centerline and resulted into visual representation of statistics of root shapes for comparison within and in between given ensembles. While this approach has been tailored for the particular problem in plant phenotyping, the method can be in principle applied to any application domain where images capture an evolving structure forming an ensemble. These could be movement trajectories of obtained from cameras of trails in public spaces for urban planning purposes, movement pattern studies of animals, such as flies, bees, ants or mice to name a few.

We have extended a recently introduced curve box plot representation with the notion of time. In addition we introduced a new visual clue to represent the statistical feasibility of the curve ensemble. We have introduced the proposed representations to the root phenotyping community and they seem to have the potential to form a standard in communicating summary of the emerging structure of the root ensemble. Last, but not least we have contributed to the phenotyping community with a new representation for roots using parameterized curves that has drastically reduced the storage requirements. While this is not a strong contribution to visualization research, it can be a game-changer in how data for root phenotyping is stored and shared within the respective research community. A similar approach can be adopted in research areas dealing with evolving linear structures where method of sharing data are still large image collections.

## Appendix A Modified Weiszfeld algorithm and *L*_1_ data depth

The geometric median (multivariate *L*_1_-median, spatial median) is the theoretical solution of the Fermat-Weber location problem. Given the sample points *X*={**x**_1_…**x**_*n*_} in an Euclidean space, the problem is to find

4 $$ \mathbf{y}=\arg \min_{\mathbf{x}}{\sum_{i=1}^{n}\left\|{{\mathbf{x}}-{\mathbf{x}}_{i}}\right\|}   $$

The modified Weiszfeld algorithm starts with initial value **y**_0_=*mean*(**x**_0_…**x**_*n*_), and until convergence the following is iterated:

5 $${} \begin{aligned} \mathbf{y}_{i+1}&= \left(1-\frac{\mathbf{w}\left(\mathbf{y}_{i}\right)}{\mathbf{r}\left(\mathbf{y}_{i}\right)}\right)^{+} \sum_{\mathbf{x}_{i}\ne \mathbf{x}}\frac{\mathbf{x}_{i}}{\lVert \mathbf{x}_{i}-\mathbf{x} \rVert} \left(\sum_{\mathbf{x}_{i}\ne \mathbf{x}}\frac{1}{\lVert \mathbf{x}_{i}-\mathbf{x} \rVert} \right)^{-1} \\ &+\min{\left(1,\frac{\mathbf{w}\left(\mathbf{y}_{i}\right)}{\mathbf{r}\left(\mathbf{y}_{i}\right)}\right)}\mathbf{y}_{i} \end{aligned}   $$

where

6 $$ \mathbf{r}(\mathbf{z})=\left\| \sum_{\mathbf{x}_{i}\ne \mathbf{z}} \frac{\mathbf{x}_{i}-\mathbf{z}}{\|\mathbf{x}_{i}-\mathbf{z} \|} \right\|  $$

and

7 $$ \mathbf{w}(\mathbf{z})= \begin{array}{ll} 1&\text{if~} \mathbf{z}=\mathbf{x}_{k}, k=1 \dots n\\ 0&\text{otherwise}. \end{array}  $$

Using **w** and **r**, the *L*_1_ data depth is defined as follows

8 $$ L_{1}D(\mathbf{x})=1-\frac{\max\left(\mathbf{r}(\mathbf{x})-\mathbf{w}(\mathbf{x}),0\right)}{n}   $$

## Appendix B Linear system for TPS fitting

The linear and non-linear coefficients of Eq. 1 are found with Least-Squares fitting, solving the system of

9 $$ A\mathbf{y}=\mathbf{b},  $$

where



Since the kernel functions are not compact, the to-be-solved linear system is badly conditioned. Therefore an iterative method is advised to use by the literature. In our experiments we used Conjugate Gradients to solve the symmetric system *A* ^*T*^ *A* **y**=*A* ^*T*^ **b**.
